# Tubal choriocarcinoma presented as ruptured ectopic pregnancy: a case report and review of the literature

**DOI:** 10.1186/s12957-020-02021-4

**Published:** 2020-09-12

**Authors:** Shengjie Xu, Xiaohong Song, Chengjuan Jin, Yanli Li

**Affiliations:** Department of Obstetrics and Gynecology, Shanghai General Hospital, School of Medicine, Shanghai Jiao Tong University, 100# Haining Rd, Shanghai, 200080 China

**Keywords:** Tubal choriocarcinoma, Ectopic pregnancy, Histopathology, Immunohistochemistry, Chemotherapy

## Abstract

**Background:**

Tubal choriocarcinoma is an extremely rare but highly malignant trophoblastic tumor, which may be either gestational or non-gestational in origin. Due to atypical clinical manifestations and symptoms similar to ectopic pregnancy, it is easily to be confused with ectopic pregnancy. In addition, inadequate understanding of this rare disease by clinicians often leads to misdiagnosis or missed diagnosis, which in turn results in delayed treatment or even tumor metastasis.

**Case presentation:**

This report summarized a case of a woman who was finally diagnosed as tubal choriocarcinoma through the follow-up of serum β hCG levels and histopathological results after undergoing salpingectomy for being misdiagnosed as ectopic pregnancy. Five courses of adjuvant chemotherapy (5-fluorouracil, actinomycin-D, vinorelbine regime) have been administered to the patient in the prevention of any recurrences. During 1-year follow-up, the patient was asymptomatic and presented no evidence of recurrence.

**Conclusions:**

Tubal choriocarcinoma is easily to be confused with ectopic pregnancy. By analyzing this case and previous related cases, we aimed to provide references for clinicians in the diagnosis and treatment of tubal choriocarcinoma.

## Background

Tubal choriocarcinoma is a kind of highly malignant trophoblastic tumor, which can be either gestational or non-gestational in origin. According to reports, the incidence of tubal choriocarcinoma is about 1.5/10^6^ [1]. The age of onset ranges from 16 to 56 years with an average of 33 years. Non-gestational tubal choriocarcinoma is extremely rare, and gestational tubal choriocarcinoma tends to occur on the basis of tubal pregnancy. In few cases, gestational tubal choriocarcinoma occurs after in vitro fertilization and fallopian tube sterilization, and even simultaneously with an intrauterine pregnancy [2].

Tubal choriocarcinoma can be easily misdiagnosed as ectopic pregnancy due to its main clinical manifestations similar to those of ectopic pregnancy, such as amenorrhea, irregular vaginal bleeding, abdominal pain, and elevated serum β hCG. Delayed treatment or even tumor metastasis may be caused if not diagnosed and intervened in time. Therefore, how to make a differential diagnosis and better understand and treat such diseases has attracted more and more attention from obstetricians and gynecologists in recent years. This article summarizes and analyzes the epidemiology, disease characteristics, clinical manifestations, diagnosis, and treatment of tubal choriocarcinoma that is easily misdiagnosed as ectopic pregnancy, with a view to provide a reference for clinicians.

## Case presentation

A 42-year-old Asian woman (gravida 5, para2) was admitted in the emergency room due to amenorrhea of 37 days, vaginal bleeding for 1 week, and intensive lower abdominal pain for half day. On examination, her vital functions were stable, blood pressure was 104/72 mmHg, and pulse rate was 83/min. Gynecological examination revealed a small amount of dark blood in the vagina and a sharp bilateral adnexal pain with rebound tenderness.

**Fig. 1:**
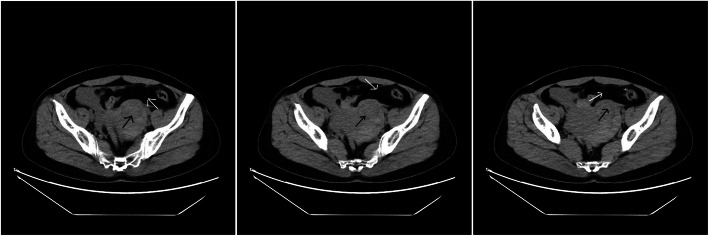
Preoperative CT scan of the abdomen with arrow mark showing adnexal mass (black) and bloody pelvic effusions (white)

On laboratory examination, leukocyte count 14,880/mm^3^, hemoglobin 10.2 g/dl, and serum β hCG > 10,000 mIU/ml were detected. CT scan revealed the presence of a low-density shadow in the left accessary area and a large amount of bloody effusions in the pelvic cavity (Fig. 1).

A woman of reproductive age (a history of abortion 13 months ago) presented with amenorrhea, vaginal bleeding, lower abdominal pain, and elevated serum β hCG level raises the suspicion of ruptured ectopic pregnancy, which always presents with similar features and was considered as the first possibility in this case. Subsequently, approximately 3 ml non-coagulated blood was extracted from the puncture of posterior fornix of vagina. Considering that persistent bleeding may be life-threatening, a diagnostic laparoscopy was carried out immediately. Upon entering the abdominal cavity, blood clot of approximately 500 ml was seen and subsequently evacuated. The uterus was normal. The right fallopian tube and ovary were normal in appearance. The left ovary was within normal limits but there was a ruptured and actively bleeding ectopic mass of approximately 6 × 5 cm at the ampulla of left fallopian tube. A left-sided salpingectomy was carried out. Finally, the mass was sent for histopathological examination and the serum β HCG levels were monitored continuously during postoperative period (Table 1).

**Table 1:** Monitoring of the serum β hCG levels during perioperative stage

Date	β hCG (mIU/mL)	Note
Pre-operation	> 10,000	No exact value
Operation day	> 10,000	No exact value
1st day after operation	8152	/
2nd day after operation	5148	/
10th day after operation	570.9	/
13th day after operation	217.0	/
14th day after operation	127.7	/
15th day after operation	95.78	FAV #1 start
21st day after operation	2.1	FAV #1 end

*FAV* 5-fluorouracil, actinomycin-D, vinorelbine

**Fig. 2:**
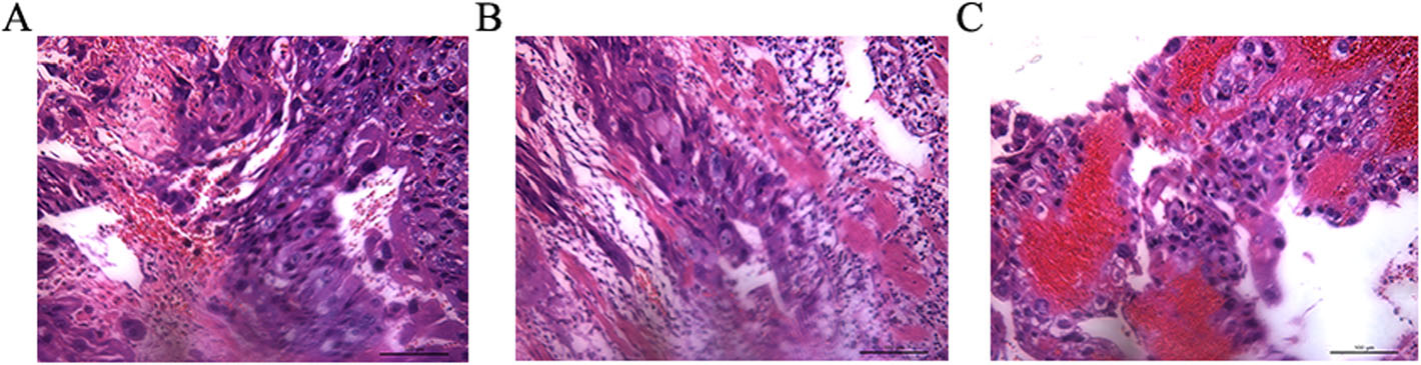
Histopathologic view of left tubal choriocarcinoma presenting the biphasic feature of mixed cytotrophoblasts and syncytiotrophoblasts (**a**) infiltrating the muscle layer of the fallopian tubal wall and the soft tissue of the adventitia (**b**). Extensive hemorrhage was present, as was necrosis (**c**). Hematoxylin and eosin (H&E) stain. × 200

**Fig. 3:**
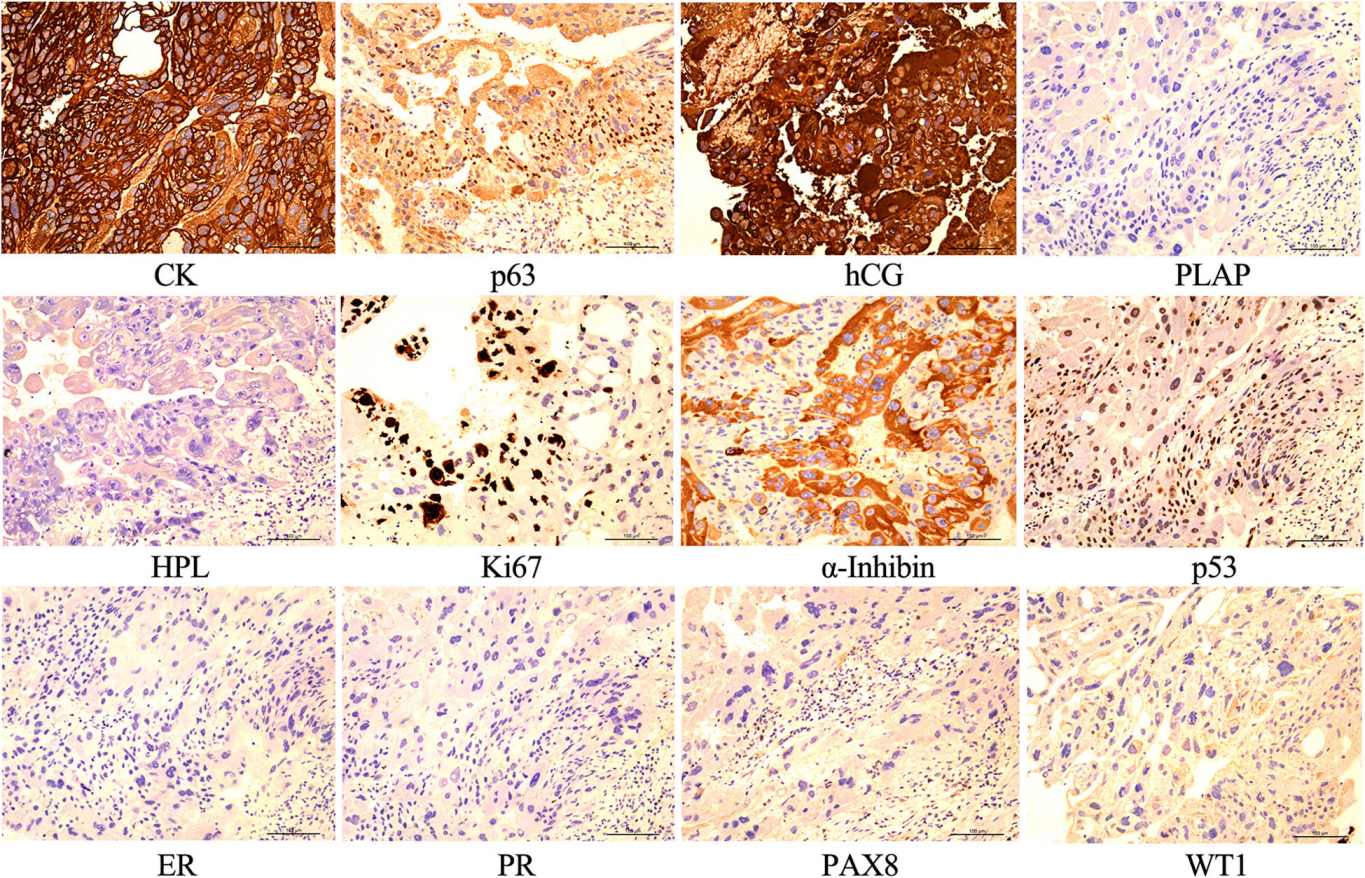
The application of immunohistochemical markers provided further support for the diagnosis of tubal choriocarcinoma (**d**). × 200

Histopathological examination revealed that cytotrophoblasts and syncytiotrophoblasts were significantly proliferated in the mass, infiltrating the muscle layer of the fallopian tubal wall and the soft tissue of the adventitia, accompanied by extensive hemorrhage and necrosis (Fig. 2). Immunohistochemical analysis was positive for β hCG, cytokeratin (CK), p63, p53, and α-inhibin and negative for human placental lactogen (HPL), placental alkaline phosphatase (PLAP), PR, ER, PAX8, and WT1, whereas Ki 67 proliferation index was 60% (Fig. 3). The final diagnosis was tubal choriocarcinoma based on these findings.

Subsequently, a metastatic workup including CT scans of the brain, chest, and abdomen and contrast-enhanced MRI of the pelvis comprehensively ruled out the possibility of systemic metastasis of the tumor. Finally, the patient was categorized into FIGO stage I with WHO prognostic score of at least 10, representing high risk. Details of the prognostic scoring were shown as follows: age—score 1 (42 years of age), antecedent pregnancy—score 1 (abortion), interval from index pregnancy—score 4 (patient had an abortion ≥ 13 months back), pretreatment β HCG value—score at least 2 (> 10,000 mIU/ml, no specific value), largest tumor size—score 2 (approximately 6 × 5 cm), site of metastasis—score 0 (none), number of metastatic lesions—score 0 (none), and previous failed chemotherapy—score 0 (none).

The serum β hCG level dropped to 95.78 mIU/ml at 15th day after operation and just before implementing adjuvant chemotherapy (5-fluorouracil, actinomycin-D, vinorelbine (FAV) regime). The normal level of β hCG was achieved after the first course of adjuvant chemotherapy (Table 1). Subsequently, four courses of consolidation chemotherapy in total were administered to the patient at intervals of 4 weeks in the prevention of any recurrences. The main adverse reactions during consolidation chemotherapy are bone marrow suppression and gastrointestinal. However, the toxicity and the side effects were tolerable after conventional symptomatic and supportive treatment.

In addition, the patient was advised to avoid pregnancy for 2 years and instructed to take effective contraceptive measures. During 1-year follow-up, the patient was asymptomatic and presented no evidence of recurrence. At present, the patient is still being followed up by performing serum β hCG testing and imaging examination at regular intervals.

## Review of the literature

The available literatures of tubal choriocarcinoma presenting as ectopic pregnancy were obtained from MEDLINE and Google Scholar. Two scholars independently screened qualified publications from the database through the content of the title and abstract. A total of 12 cases were extracted from the databases, and the disease characteristics, clinical manifestations, treatment, and outcome of tubal choriocarcinoma were summarized in Table 2.

**Table 2:** Summary of cases of tubal choriocarcinoma

Author	Age	Clinical presentation	β hCG (mIU/ml)	Tumor size (cm)	Surgery	Chemotherapy	Outcome
Mehrotra et al. [2]	30	Abdominal pain, fever, fatigue, tachycardia, and palpable mass 1 month after first trimester abortion	326,100	14 × 16	Left-sided salpingoophrectomy	EMA-CO	No evidence of recurrence
Mundkur et al. [3]	26	Mass abdomen, pain in the lower abdomen, vomiting, loss of weight, and loss of appetite	151,545	10 × 10 × 5	Right adenexectomy with partial infracholicomentectomy	EMA-CO	No evidence of recurrence
Rettenmaier et al. [4]	32	Abdominal cramping	4759	3.5 × 1.7 × 2.8; 4.3 × 1.3 × 4.4	Left-sided partial salpingectomy	Refused chemotherapy	Refused follow-up
Karaman et al. [5]	31	Amenorrhea, left lower abdominal pain, and fatigue	29,251.4	4 × 3	Left-sided complete salpingectomy	MTX	No evidence of recurrence
Cianci et al. [6]	30	Left lower abdominal pain	24,474	8	Left-sided salpingoophrectomy	EMA-CO	No evidence of recurrence
Boynukalin et al. [7]	38	Abdominal pain and vaginal bleeding	> 15,000	None	Right-sided salpingectomy	None	None
Gálvez et al. [8]	33	Intense pain in the right iliac cavity and limited genital bleeding	142.1	3.73	Right-sided salpingectomy	VCR, ActD, MTX, LV	No evidence of recurrence
Lee et al. [9]	31	Dyspnea and blood-tinged sputum	228,300	7 × 6 × 4	Tumorectomy with left salpingectomy and infracolic omentectomy	EMA-CO	No evidence of recurrence
Butler et al. [10]	24	Vaginal bleeding, lower abdominal pain, and amenorrhea	15,000	2.6	Left-sided salpingectomy	MTX	No evidence of recurrence
Lin et al. [11]	38	Pregnancy of unknown location over 9 months following ovarian induction	267,836	5	Excision of the right ovarian cyst and the left uterine tube	MTX	No evidence of recurrence
Ibrahim et al. [12]	34	A positive urine test and respiratory distress	752,601	None	Left-sided salpingectomy and wedge resection of the broad ligament	None	Died of lung metastasis of tubal choriocarcinoma
Nakayama et al. [13]	26	Genital bleeding and a persistently high level of β hCG	9903	6.4 × 1.4	Right-sided salpingectomy	None	No evidence of recurrence

*EMA-CO* etoposide, methotrexate, actinomycin-D, cyclophosphamide, oncovin; *MTX* methotrexate; *ActD* actinomycin-D; *LV* leucovorin; *VCR* vincristine

## Discussion

Tubal choriocarcinoma is a rare but aggressive ectopic choriocarcinoma and may be either gestational or non-gestational in origin [14]. Regardless of gestational or non-gestational tubal choriocarcinoma, the common feature is that early metastasis and spread of the cancer are prone to occur, of which lung metastasis ranks the first place, followed by para-uterine metastasis [9, 12].

Patients with tubal choriocarcinoma are rare and often have similar clinical symptoms to those of tubal ectopic pregnancy including amenorrhea, vaginal bleeding, pelvic pain, and increased serum β hCG [2, 15]. Therefore, diagnostic errors always occurred. In addition, the respiratory symptoms of advanced tubal choriocarcinoma patients with lung metastasis were often ignored, leading to delayed therapy and disease progression [9, 12]. Therefore, it is essential to exclude choriocarcinoma when diagnosing ectopic pregnancy, especially when patients are accompanied by respiratory distress, bloody sputum, and other respiratory symptoms.

It is important to distinguish between tubal choriocarcinoma and tubal ectopic pregnancy through dynamic monitoring of serum β hCG levels, diagnostic laparoscopy, and histopathological examination. In general, the serum β hCG of patients with tubal choriocarcinoma tended to abnormally elevate within a short time after amenorrhea, while that of patients with tubal ectopic pregnancy rarely exceeds 10,000 mIU/ml [14]. In some cases, the serum hCG levels of patients after surgery were not significantly reduced or even continuously increased. On this occasion, the tubal choriocarcinoma, even very rare, should be kept in mind. Diagnostic laparoscopy has a unique advantage in dealing with life-threatening intra-abdominal bleeding caused by either ruptured ectopic pregnancy or tubal choriocarcinoma. Most importantly, histopathological specimens used for the final diagnosis could be obtained from diagnostic laparoscopy. In addition, it is essential to carefully dissect and inspect the excised lesions during the operation. When no villous structures are visually observed in the excised lesions, it is strongly recommended to execute intraoperative frozen section biopsy for the differential diagnosis between ectopic pregnancy and tubal choriocarcinoma. Histopathology is the golden standard for diagnosing choriocarcinoma. The typical histological characteristics of tubal choriocarcinoma are columns of hypertrophic and poorly differentiated trophoblastic cells without villous structures and the invasion of muscular tissue with extensive hemorrhage and necrosis [2, 6].

Adjuvant chemotherapy following salpingectomy is essential and effective for the treatment of tubal choriocarcinoma. Currently, the chemotherapy regimens for tubal choriocarcinoma were selected with reference to the regimens used in the treatment of trophoblastic tumor. In general, patients of low-risk are treated with single-agent chemotherapy, while patients of high-risk are treated with combined chemotherapy. At present, the level of serum β hCG is widely used as the main criterion for judging the therapeutic effect of tubal choriocarcinoma. But even when the serum hCG reaches the normal level, consolidation chemotherapy is still recommended in prevention of clinical recurrences [2, 6, 14]. Generally, 1 to 2 courses of consolidation chemotherapy are recommended for low-risk patients and 2 to 4 courses for high-risk patients. Life-long serum β hCG monitoring and imaging examination is essential for patients with tubal choriocarcinoma as there is still no clear guideline indicating when to stop monitoring [16].

## Conclusion

When diagnosing ectopic pregnancy, any other disease of tubal origin like tubal choriocarcinoma should be kept in mind for differential diagnosis. Careful examinations of pathologic specimens and postoperative monitoring of β hCG titers are emphasized to avoid misdiagnosis of ectopic tubal choriocarcinoma, although it is a rare condition. Apart from complete surgical resection, principles for the management of tubal choriocarcinoma also include postoperative adjuvant chemotherapy, imaging follow-up, and lifetime β hCG monitoring in order to avoid any risk of metastasis and recurrences.

## Data Availability

The datasets used and/or analyzed during the current study are available from the corresponding author on reasonable request.

## Abbreviations

FAV: 5-Fluorouracil, actinomycin-D, vinorelbine
hCG: Human chorionic gonadotropin
CK: Cytokeratin
HPL: Human placental lactogen
PLAP: Placental alkaline phosphatase
EMA-CO: Etoposide, methotrexate, actinomycin-D, cyclophosphamide, oncovin
MTX: Methotrexate
ActD: Actinomycin-D
LV: Leucovorin
VCR: Vincristine
